# Assessment of colonic contents in patients with chronic constipation using MRI

**DOI:** 10.3164/jcbn.17-104

**Published:** 2018-03-17

**Authors:** Yumi Inoh, Kenji Kanoshima, Kanji Ohkuma, Akiko Fuyuki, Shiori Uchiyama, Hidenori Ohkubo, Takuma Higurashi, Hiroshi Iida, Takashi Nonaka, Koji Fujita, Akihiko Kusakabe, Masahiko Inamori, Kazumasa Hiroishi, Hajime Nagase, Atushi Nakajima, Taro Takahara

**Affiliations:** 1 Department of Hepatology and Gastroenterology, Yokohama City University School of Medicine, Yokohama, Kanagawa 236-0004, Japan; 2 Department of Gastroenterology, Yokohama Rosai Hospital, Yokohama, Kanagawa 222-0036, Japan; 3 Department of Medical Education, Yokohama City University School of Medicine, Yokohama, Kanagawa 236-0004, Japan; 4 Department of General Medicine, Yokohama City University School of Medicine, Yokohama, Kanagawa 236-0004, Japan; 5 Department of Gastroenterology, Shin-Yurigaoka General Hospital, Kawasaki, Kanagawa 215-0026, Japan; 6 Department of Biological Engineering, Tokai University, School of Biological Engineering, Isehara, Kanagawa 259-1193, Japan

**Keywords:** constipation, MRI, GSRS, nausea and diarrhea

## Abstract

Although chronic constipation is common, colonic functional evaluating tests are uncommon. This study examines whether chronic constipation and gastrointestinal symptoms are correlated with the lateral diameter of the colon measured from MRI images. We included chronic constipation patients in a prospective, cross-sectional study using MRI at three centers. We divided 3D MRI colorectal images into 6 segments using with specified sequences and selected the maximum luminal diameter from each segment. We used the GSRS questionnaire to evaluate gastrointestinal symptoms. We evaluated the correlation between luminal diameters and GSRS scores. We found the following positive correlations: descending colon and unsatisfactory defecation symptoms; sigmoid colon and diarrhea; and rectum and constipation. The sum and ratio of the ascending and sigmoid colon diameters correlated with nausea and diarrhea. The sum of the transvers to the sigmoid colon diameter also correlated with nausea and diarrhea. The sum of all segment diameters correlated with nausea and constipation. In conclusion, we showed cross-sectional study of colonic MRI correlate with gastrointestinal symptoms. MRI might be useful for colonic motility evaluations to determine appropriate constipation treatments (Clinical trial registry number UMIN 000021274).

## Introduction

Chronic constipation is a common disorder; 2.9% of Japanese men and 4.9% of Japanese women describe themselves as constipation sufferers. In America, chronic constipation occurs in approximately 15% of the population and increases with age.^(1,2)^ The Rome III diagnostic criteria of functional gastrointestinal disorders define at least 2 physiological dysfunctions for chronic constipation: delayed colon transit and defecation disorder.^(3)^ However, there are few stool distribution examinations, and they are not universally applied. For example, in some places, radiopaque markers are used to assess colon transit time, but in Japan, radiopaque tests are not permitted by the Ministry of Health, Labour and Welfare.

Magnetic resonance imaging (MRI) has many advantages, including no requirement for ionizing radiation and good soft tissue contrast, speed, resolution, and multi-planar capability.^(4)^ Furthermore, because this technology is radiation-free, it does not affect fertility in young women.

Several studies have assessed gastrointestinal motility disorders using MRI. For colon motility, Pritchard *et al.*^(5)^ used serial MRI to analyze the volume of the undisturbed colon in healthy volunteers and in patients with diarrhea-predominant irritable bowel syndrome. Internationally, however, no studies have evaluated chronic constipation using MRI.

In 2000, Takahara *et al.*^(6)^ reported an MRI sequence focusing on the stool distribution in the colon that could diagnose constipation in a Japanese journal. They included 101 patients who received MRI for any reason except colon disease and examined correlations with constipation symptoms. Patients who had last defecated more than 2 days before exhibited significantly larger lateral distal transverse colon and sigmoid colon diameters than patients who had defecated within 1 day (*p*<0.01). They also reported that patients who considered themselves constipation sufferers showed the same results (*p*<0.05).

However, this report is the most recent on this topic. Therefore, the current study examines whether chronic constipation and gastrointestinal symptoms are correlated with the lateral diameter of the colon measured from MRI images.

## Methods

### Study design

This study was prospective and cross-sectional in design, using MRI at three centers including Yokohama City University Hospital, Yokohama Rosai Hospital and Shin-Yurigaoka General Hospital.

### Subjects

We included patients who described themselves as chronic constipation sufferers that showed no evidence of mechanical obstruction, such as a colorectal cancer, regardless of whether they fulfilled the Rome III diagnostic criteria for chronic constipation because constipation is not clearly defined in Japan. Patients had visited one of the three hospitals from March 2016 to March 2017. A total of 20 patients who agreed to participate in the study [13 females and 7 males, 42.6 (SD 18.4) and 60.4 (SD 17.4) years old, respectively] were selected (Table 1).

We excluded patients diagnosed with organic digestive diseases in the last year, those with contraindications to MRI, and patients who had undergone gastrointestinal excision, including appendectomy. If a patient was inappropriate for the trial due to severe heart failure, hepatic failure, renal failure, or terminal cancer, they were not recruited.

Food and drug limitations, including laxatives, were not imposed on the subjects, who were instructed to record the time of their last meal and any drug use. They also recorded their bowel habits 7 days before the examination.

### Rating of gastrointestinal symptoms

Participants were asked to complete a modified gastrointestinal symptoms rating scale (GSRS) questionnaire to evaluate the presence and severity of general GI symptoms during the past week. The GSRS is a 15-item questionnaire with a 7-point Likert scale (1 = none, 2 = minor, 3 = mild, 4 = moderate, 5 = moderately severe, 6 = severe and 7 = very severe discomfort). It was originally constructed as an interview-based rating scale to evaluate a wide range of GI symptoms (S. I. Svedlund J) and was later modified to be a self-administered questionnaire (Dimenäs E). The items are grouped into five subscales, including “*abdominal pain*” (three items), “*reflux”* (two items), “*indigestion*” (four items), “*diarrhea*” (three items), and “*constipation*” (three items) syndromes.^(7)^

### Magnetic resonance imaging

All of the subjects were examined using a 1.5-T MRI system from one of three MR vendors [(1) Magnetom Symphony, Siemens Healthcare, Erlangen, Germany; (2) Signa HDxt, GE Healthcare, Milwaukee, United States; and (3) Excelart Vantage, Toshiba Medical, Tokyo, Japan].

Three-dimensional T1-weighted gradient-echo imaging (3D-VIBE, 3D-LAVA and 3D-T1-FFE) was performed with the following sequence parameters with maximum effort using common parameter values across the scanners: repetition time (TR)/echo time (TE) 4.5–6.3/1.9–2.4 ms, flip angle 10–12 degree, image acquisition in the coronal plane, slice thickness 3–4 mm, number of slices 40–56, field of view (FOV) 400 mm^2^, acquisition matrix 208–256, 1 signal average, actual pixel size 1.56–1.92 × 1.56–2.40 mm^2^, parallel imaging factor of 2, image acquisition under breath-holding, total scan time 18–26 s.

### Data collection

Image analysis was performed using a clinical general image viewer rather than special software. We divided each colorectal image into 6 segments (*ce*: cecum, *a*: ascending colon, *t*: transverse colon, *d*: descending colon, *s*: sigmoid colon and *re*: rectum). We identified each of segments as follows. The cecum was the terminal end of the ilium. The ascending colon was the segment between the end of the ileum and the hepatic flexure. The transverse colon was the segment between the hepatic flexure and the splenic flexure. The descending colon was the segment from the splenic flexure to the caudal side of the line of both iliac spines. The sigmoid colon was the segment from the caudal side of the line of both iliac spines to the oral side of the recto-sigmoid. The rectum was the terminal end of the colon.

**Fig. 1:**
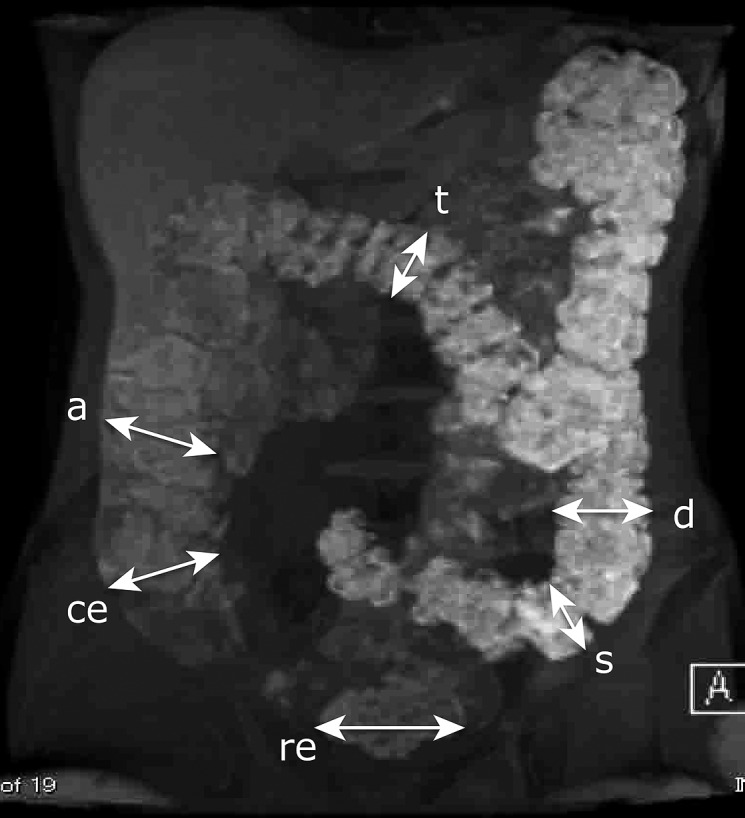
An auxiliary line perpendicular to the long axis of the selected loops was drawn along 10 points in each section, and the maximum diameter of each section was selected. We measured these diameters primarily using 3D images with partial supplementation by 2D images. *ce*, cecum; *a*, ascending colon; *t*, transverse colon; *d*, descending colon; *s*, sigmoid colon; *re*, rectum.

An auxiliary line perpendicular to the long axis of the selected loops was drawn along 10 points in each segment to select the maximum diameter of each segment. We measured these diameters mainly using 3D images with partial supplementation from 2D images (Fig. 1). We named these maximum diameters *ce*, *a*, *t*, *d*,* s* and *re* as acronyms for each segment.

We evaluated the correlation between each maximum diameter of *ce*, *a*, *t*, *d*, *s* and* re* and each score on the GSRS. In addition, we evaluated the sum of all diameters, *ce* + *a* + *t* + *d* + *s* + *re*. Because Takahara *et al.*^(6)^ evaluated diameters from the distal transverse colon to the sigmoid colon as well as the sum of the diameters in the distal descending colon and the sigmoid colon, we evaluated *d* + *s* and *t* + *d* + *s*.

In general, the ascending colon and the sigmoid colon serve as stool storage. Therefore, we also evaluated the sum and ratio of *a* + *s* and *s/a*.

We named the time from the last meal to examination *x* (hours) and the time of last defecation until examination as *y* (hours). No correlations were found between *x* and each diameter. A correlation was observed only between* y* and *s* [the correlation coefficient between *y *and *s* was 0.513 (*p* = 0.025)]. We considered this an unavoidable bias because the sigmoid colon diameter is small when the time from the last defecation is short. However, no correlations were observed between laxative use and each diameter.

### Statistical analysis

In this study, statistical evaluation was performed with the Spearman’s rank correlation coefficient. The level of significance was set at a *p* value of <0.05. We considered a possible correlation if the correlation coefficient was >0.4. All of the statistical analyses were performed with EZR (Saitama Medical Centre, Jichi Medical University, Saitama, Japan), which is a graphical user interface for R (The R Foundation for Statistical Computing, Vienna, Austria). More precisely, it is a modified version of R commander designed to add statistical functions that are frequently used in biostatistics.^(8)^

### Ethics

The study (clinical trial registry number UMIN 000021274) was conducted in accordance with the principles of the Declaration of Helsinki. Prior to study initiation, written informed consent was obtained from all of the participants. This study was approved by the Ethics Committee of Yokohama City University School of Medicine.

## Results

We evaluated the correlations between each diameter *ce*, *a* and *t*, and each score on the GSRS, but no correlations were found. A positive correlation was observed between *d* and scores for GSRS15 (the feeling of unsatisfactory defecation) and total GSRS scores. A positive correlation was found between *s* and scores for GSRS11 (diarrhea) and “*diarrhea*” as one of the subscales. A positive correlation was observed between the size of the rectum and scores for GSRS10 (constipation).

Positive correlations were identified between *d* + *s* and scores for GSRS5 (nausea), GSRS11, “*diarrhea*” as one of the subscales, and total GSRS scores. The sum of the diameters *t* + *d* + *s* showed positive correlations with scores for GSRS11 and “*diarrhea*” as one of the subscales.

The sum of the diameters *a* + *s* showed positive correlations with scores for both GSRS5 and GSRS11. The ratio of diameters, namely *s/a*, demonstrated positive correlations with the scores for GSRS5, GSRS11 and “*diarrhea*” as one of the subscales.

The sum of all of the diameters, *ce* + *a* + *t* + *d* + *s* + *re*, exhibited positive correlations with the scores for GSRS5 and GSRS10 (Table 2).

## Discussion

In this study, we evaluated patients with constipation using MRI. In Japan, a previous observational study using MRI showed that the diameter of the colon not including the cecum and the rectum was larger in a group of constipated patients than in a group of non-constipated patients. Therefore, we formulated the hypothesis that a larger colon diameter corresponds to stronger constipation symptoms.

In our study, no correlations were observed between colon segments and GSRS score. On the GSRS, a strong feeling of unsatisfactory defecation correlated with a large diameter of the descending colon. Strong constipation feelings correlated with a large rectum diameter, although rectum diameter was not correlated with constipation in a previous study.

Bockus gastroenterology by Roth *et al.*^(9)^ described distention of the descending colon of producing a sensation of gas and bloating, whereas distention of the rectum results in a feeling of urgency.^(10)^ This description suggests that if the sigmoid colon diameter is large, patients feel the urge to defecate, but if the descending colon diameter is large, patients experience an unnecessary urge to defecate, leading to patients experiencing unsatisfactory defecation easily.

In Rome III, each segment of the colon is described as follows. The ascending colon has a neuromuscular mechanism that accommodates the stomach, allowing the cecum and ascending colon to fill without excess intraluminal pressure, which would induce anterograde and retrograde peristalsis. If stool fills the transverse colon, anterograde peristalsis in the ascending colon is regulated by a feedback mechanism. The transverse colon is a very important segment, because it holds stagnated stool for 24 h and removes water and electrolytes. The propagation of a large migrating contraction from the transverse colon to the descending colon transfers stool to the sigmoid colon. Together, the sigmoid colon and the rectum can store approximately 500 ml of material.

These facts suggest that feces becomes stagnant in either the ascending or sigmoid colon in humans with normal habits. Therefore, if both the ascending and sigmoid colon have large diameters, this might elicit strong symptoms of constipation.

However, in our study, neither the ascending nor the sigmoid colon diameter correlated with constipation symptom scores. Instead, these measurements correlated with diarrhea scores; specifically, *s/a* was positively correlated with scores regarding diarrhea. In other words, a larger sigmoid colon to ascending colon ratio corresponded to stronger symptoms of diarrhea. However, this result seems to contradict the relationship between a short stool storage duration in the transverse colon and an insufficient dehydration of feces resulting in diarrhea. Simultaneously, when wall extension of the sigmoid colon leads to serious contradictions, patients feel a frequent urge to defecate rather than bloating. Viewed another way, patients with a large quantity of feces in the sigmoid colon often use laxatives and paradoxically develop diarrhea symptoms despite a constipation etiology.

However, the diameter sum of all segments correlated with nausea and constipation scores, suggesting that patients with large amounts of feces in all segments of the colon feel symptoms of constipation easily. Large quantities of feces can cause upper gastrointestinal tract symptoms, including nausea.

This study included patients with constipation symptoms, and the results suggest that these patients not only have constipation symptoms but also other gastrointestinal symptoms if large fecal quantities are retained in the colon.

Imaging using MRI sequences might be useful for colonic motility evaluations to determine the appropriate treatment for constipation, because MRI is a common examination tool in clinical practice worldwide.

This study had several limitations. First, the measurement points of the colon diameter were not predefined and were manually measured. To reduce errors, we measured diameters at 10 points in each segment and selected the maximum diameter, but the results are still subjective. Second, we could not order MRI tests at specified times because tests were being conducted for other patients at the same time. Therefore, we could not match the times from the last meal and last defecation. To overcome this weak point, we analyzed the correlations between colon diameters and the time from the last meal and the last defecation, but no significant correlations were observed. If possible, measurements taken at the same time points are desirable.

Third, the MRI models were different in each hospital. To reduce errors between the models, we asked Dr. Takahara, who published a previous study, to make subtle adjustments to the MRI machine at each center. We do not think that MRI model standardization is necessary because of the universal use of MRI, but software for measuring and analyzing colon diameters and volumes is needed. We hope that MRI conditions will be normalized in the future.

We evaluated correlations between fecal storage and gastrointestinal symptoms by analyzing colon diameters by MRI in constipated patients. Our study suggests that patients with large sigmoid colon diameters had strong symptoms of diarrhea rather than constipation. If patients have large diameters in all colon segments, they have stronger constipation and upper gastrointestinal symptoms.

## Author Contributions

Study concept and design; Yumi Inoh, Kenji Kanoshima, Kanji Ohkuma, Hiroshi Iida, Takashi Nonaka, Akihiko Kusakabe, Masahiko Inamori, Atushi Nakajima and Taro Takahara.

Acquisition of data; Yumi Inoh, Kenji Kanoshima, Kanji Ohkuma, Akiko Fuyuki, Shiori Uchiyama, Hidenori Ohkubo, Takuma Higurashi, Hiroshi Iida, Kazumasa Hiroishi, Hajime Nagase, Masahiko Inamori and Taro Takahara.

Analysis and interpretation of data; Yumi Inoh, Masahiko Inamori and Taro Takahara

Drafting of the manuscript; Yumi Inoh, Hiroshi Iida, Koji Fujita, Masahiko Inamori and Taro Takahara.

Critical revision of the manuscript for important intellectual content; Yumi Inoh, Koji Fujita, Masahiko Inamori and Taro Takahara.

Statistical analysis; Yumi Inoh and Masahiko Inamori.

Obtained funding; Koji Fujita, Masahiko Inamori and Atushi Nakajima.

Administrative, technical, or material support; Yumi Inoh and Taro Takahara

Study supervision; Kazumasa Hiroishi, Hajime Nagase, Masahiko Inamori, Atushi Nakajima, and Taro Takahara.

## Figures and Tables

**Table 1:** Patient characteristics

	Total (Male/Female)
Number	20 (7/13)
Age (year)	
Average	48.85 (60.4/42.6)
SD	18.05 (17.4/18.4)
Maximum	79 (79/77)
Minimum	32 (32/22)
Bristol scale	
Average	3.975
Median	4
SD	1.59
Maximum	6
Minimum	1

**Table 2:** The correlations between each diameter on the MRI and each score on the GSRS

	GSRS total	Diarrhea	Constipation	GSRS5	GSRS10	GSRS11	GSRS14	GSRS15
*ce*	0.18	0.21	0.13	0.31	0.28	0.18	0.081	–0.099
	(*p* = 0.46)	(*p* = 0.37)	(*p* = 0.59)	(*p* = 0.18)	(*p* = 0.24)	(*p* = 0.46)	(*p* = 0.73)	(*p* = 0.68)
*a*	0.16	0	0.26	0.27	0.3	0.02	–0.14	0.13
	(*p* = 0.52)	(*p* = 1)	(*p* = 0.26)	(*p* = 0.26)	(*p* = 0.20)	(*p* = 0.93)	(*p* = 0.56)	(*p* = 0.59)
*t*	–0.063	–0.03	0.1	–0.049	0.278	–0.097	–0.059	–0.092
	(*p* = 0.79)	(*p* = 0.90)	(*p* = 0.66)	(*p* = 0.84)	(*p* = 0.24)	(*p* = 0.68)	(*p* = 0.81)	(*p* = 0.70)
*d*	*0.48	0.2	0.44	0.29	0.32	0.026	0.22	*0.48
	(*p* = 0.033)	(*p* = 0.39)	(*p* = 0.050)	(*p* = 0.22)	(*p* = 0.16)	(*p* = 0.91)	(*p* = 0.34)	(p = 0.033)
*s*	0.32	*0.50	0.12	0.57	0.25	*0.62	0.44	0.045
	(*p* = 0.16)	(*p* = 0.024)	(*p* = 0.61)	(*p* = 0.083)	(*p* = 0.29)	(*p* = 0.004)	(*p* = 0.053)	(*p* = 0.65)
*re*	0.34	0.24	0.39	0.33	*0.46	0.17	0.069	0.15
	(*p* = 0.15)	(*p* = 0.31)	(*p* = 0.088)	(*p* = 0.15)	(*p* = 0.043)	(*p* = 0.47)	(*p* = 0.77)	(*p* = 0.54)

*d* + *s*	*0.48	*0.49	0.25	*0.58	0.29	*0.51	0.42	0.18
	(*p* = 0.033)	(*p* = 0.028)	(*p* = 0.28)	(*p* = 0.007)	(*p* = 0.21)	(*p* = 0.021)	(*p* = 0.065)	(*p* = 0.46)
*t* + *d* + *s*	0.43	*0.45	0.27	*0.56	0.35	*0.46	0.37	0.14
	(*p* = 0.060)	(*p* = 0.044)	(*p* = 0.26)	(*p* = 0.010)	(*p* = 0.13)	(*p* = 0.039)	(*p* = 0.11)	(*p* = 0.55)
*ce* + *a* + *t* + *d* + *s* + *re*	0.41	0.35	0.37	*0.52	*0.45	0.32	0.21	0.17
	(*p* = 0.070)	(*p* = 0.13)	(*p* = 0.11)	(*p* = 0.018)	(*p* = 0.045)	(*p* = 0.173)	(*p* = 0.38)	(*p* = 0.46)
*a* + *s*	0.36	0.39	0.26	*0.59	0.34	*0.46	0.31	0.16
	(*p* = 0.12)	(*p* = 0.086)	(*p* = 0.28)	(*p* = 0.006)	(*p* = 0.14)	(*p* = 0.041)	(*p* = 0.19)	(*p* = 0.49)
*s/a*	0.26	*0.52	0.015	*0.48	0.19	*0.67	*0.51	–0.07
	(*p* = 0.27)	(*p* = 0.019)	(*p* = 0.95)	(*p* = 0.032)	(*p* = 0.43)	(*p* = 0.022)	(*p* = 0.022)	(*p* = 0.77)

Table 2 shows Spearman’s rank correlation coefficients (*p* value). *p value <0.05. “GSRS Total” means total score of GSRS. “Diarrhea” means one of the GSRS subgroups (GSRS11 + 12 + 14). “Constipation” means one of the GSRS subgroups (GSRS10 + 13 + 15). GSRS5 means nausea. GSRS10 means constipation. GSRS11 means diarrhea. GSRS14 means defecation urgency. GSRS15 means feeling of unsatisfactory defecation.

## Abbreviations

FOV: field of view
GSRS: gastrointestinal symptoms rating scale
MRI: magnetic resonance imaging
TE: echo time
TR: repetition time
